# Supplementary material for: Fine-scale distribution of malaria mosquitoes biting or resting outside human dwellings in three low-altitude Tanzanian villages
Source: PLoS One. 2021 Jan 28;16(1):e0245750. doi: 10.1371/journal.pone.0245750 (PMC7842886; doi:10.1371/journal.pone.0245750)

**Fig. S1.4** – Map of predicted hotspots of female mosquitoes’ density and presence of **artificial waterbodies** in the sampled grid cells in three villages in Kilombero Valley, South-Eastern Tanzania. a) *Anopheles arabiensis*, b) *Anopheles funestus*, c) other *Anopheles*.


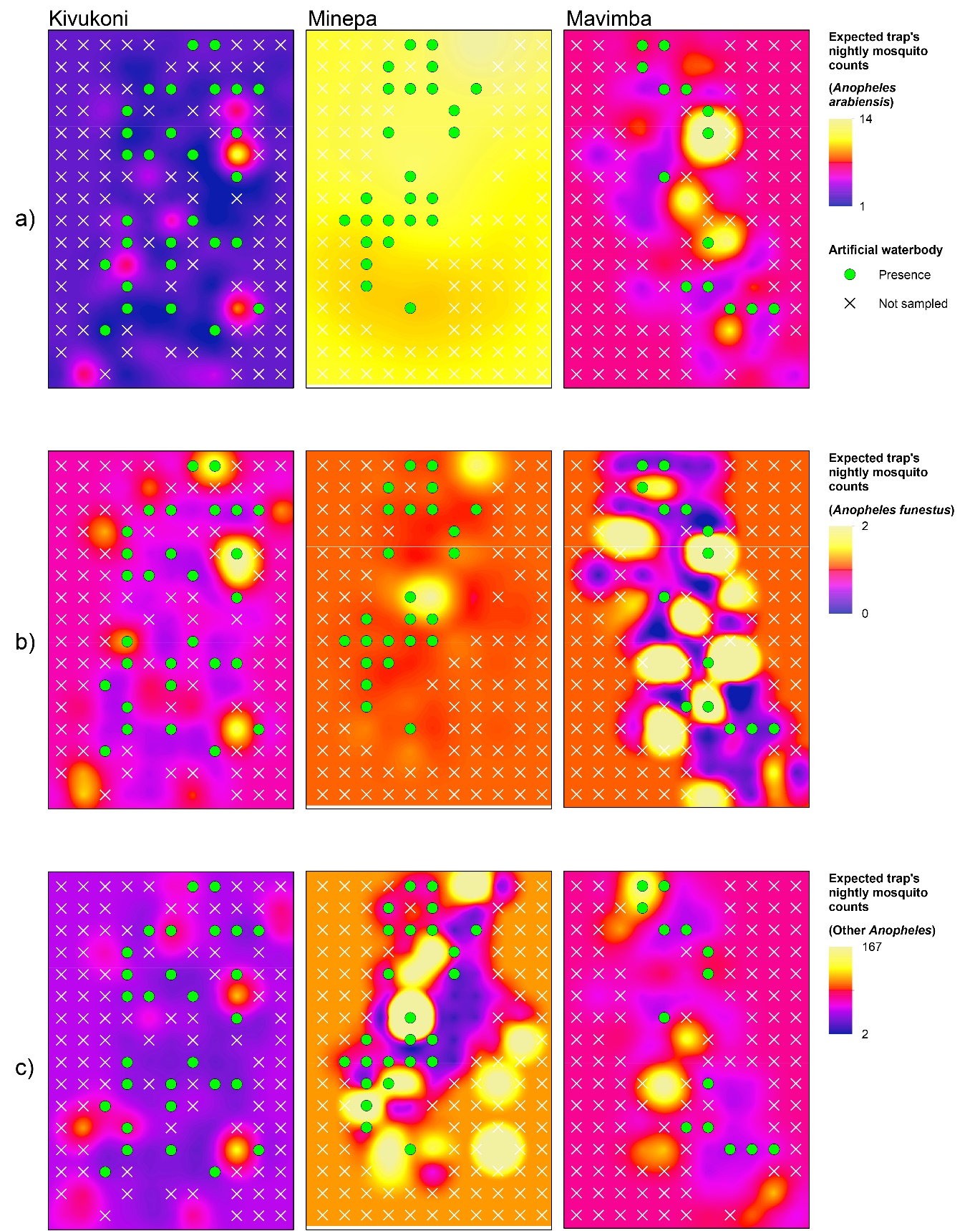

Supplement: S4 Fig — a) Anopheles arabiensis, b) Anopheles funestus, c) other Anopheles. (DOCX) [file pone.0245750.s004.docx]
