# Supplementary material for: Fine-scale distribution of malaria mosquitoes biting or resting outside human dwellings in three low-altitude Tanzanian villages
Source: PLoS One. 2021 Jan 28;16(1):e0245750. doi: 10.1371/journal.pone.0245750 (PMC7842886; doi:10.1371/journal.pone.0245750)
Supplement: Attachment — Submitted filename: Reviewrs comments_MRV_outdoo_surve_Nov_2020.docx [file pone.0245750.s018.docx]

24^th^ November 2020

Dear Editor, PLOS ONE

**Re: Research paper re-submission (Manuscript code: PONE-D-20-16651)**

We would like to thank you and the reviewers for constructive comments and for offering us the opportunity to resubmit a revised version of our paper entitled “Fine-scale distribution of malaria mosquitoes biting or resting outside human dwellings in three low-altitude Tanzanian villages”.

With regards to reviewer #3’s main concern (i.e. choice of traps that might not be the best to study outdoor transmission), we have provided further clarification on the rationale behind the choice of Suna and Resting bucket traps, including key references to justify our experimental design in this specific ecological setting.

With regards to the reviewer’s second concern (i.e. pooling the other Anopheles species or keeping them separated), we have discussed this extensively between ourselves, and the large number of field collaborators taking part in the extended project. Given the low contribution to malaria transmission given by non-*An. arabiensis* and non-*An. funestus* vectors, we decided not to put too much emphasis on the other Anopheles species. In addition, if we should consider each species separately, the length of the manuscript would increase substantially, and would pull the reader’s attention out of the main focus of our manuscript (i.e. focusing on vectors known to be responsible for malaria transmission in our study area).

We have now included further clarification with respect to the previous points, by modifying some paragraphs in the data analysis and discussion sections, and by adding mor relevant references.

Finally, we made several minor changes (and typos corrections) to further improve the reading flow. We believe our manuscript has substantially improved.

Please find our detailed point-by-point response to each reviewer’s report. We have highlighted in yellow the specific changes/additions made, in the manuscript main body.

Once again thank you very much for your support and hope to hear from you.

Yours sincerely

Arnold

[ammbando@ihi.or.tz](mailto:ammbando@ihi.or.tz)

**Response to reviewer’s comments**

**Reviewer # 3**

**Comments 1**: The choice of traps might not be the best to study outdoor transmission, as raised by the authors themselves on lines 387-389, because host-seeking traps and humans in the vicinity introduced a bias, An. funestus being highly anthropophilic, this species will avoid the traps to feed on humans. This is particularly obvious in Table 1a in which only 155 specimens of An. funestus s.l. where collected compared to 1556 specimens of An. arabiensis. Is this small number due to the traps that were not appropriate for this species or to its low density? Therefore, the choice of the 2 types of traps should be better explained.

**Response:** Thank you very much for this constructive comment. The higher number of *Anopheles arabiensis* vector abundance over *Anopheles funestus* reflects the specific ecological setting of our study area. In Kilombero valley previous malaria vector surveillance works caught significant higher number of *An. arabiensis* as compared to *An. funestus* mosquitoes (see for example Kaindoa,.et al 2017) [1]. However, about 80% of malaria transmission occurring in the study area are dominated by *An. funestus* and only 20% was due to *An. arabiensis* [1]. Apart from the difference in vector composition of these primary vectors, feeding and resting behaviours shown by these two vectors are highly different, with *An. funestus* showing preference for indoor biting and resting and *An. arabiensis* outdoor.

In our study we based the choice of our traps (Suna-traps, and Resting bucket (RBu)) based on the results of several other studies focusing on outdoor sampling in both malaria and non-malaria vectors [2-4]. We have now expanded the method section providing further justification for the choice the traps. Please see lines 159 - 164 in the revised version. Furthermore, we could have used HLC method, however this sampling methods is currently not recommended due to ethical concerns of exposing volunteers to mosquito bites.

**Comment 2**: Seven mosquito taxa have been collected during the 12 months collection (Table 1b). However, the manuscript is focusing on 2 species only, An. arabiensis and An. funestus. The third category is named "Other Anopheles". The paper would gain in clarity and robustness if the other species were also taken into consideration, especially as the data are already available (Table 1b). When possible, the spatio-temporal analysis should be more specific, including for instance An. zeimanni which was collected in high numbers (n=5607 specimens), 5 times more than An. arabiensis.

**Response**: Thank you very much for pointing this out. We agree that splitting the other malaria vectors by each species would be of interest. However, please note that the relative contribution of non- *An. arabiensis* and non-*An. funestus* vectors to malaria transmission in this specific ecological setting is low (as we also highlighted in the revised introduction section). Therefore, we deliberately decided not to put too much emphasis on the other Anopheles species, to avoid distracting the reader from the main focus of our work (i.e. outdoor biting and resting of vectors responsible for malaria transmission). Furthermore, please note that if we should expand the analysis to each separate species, the length of our manuscript (and figures and tables) would increase substantially at the cost of losing conciseness

**Additional comment**: They are also a good number of minor corrections and comments that have been included into the attached manuscript for its improvement (see attached file). For instance, they are some statements that need further explanation to increase their understanding.

**Response**: We really appreciate your additional efforts in providing these editorial suggestions. We have revised the manuscript accordingly. Please find the revised edits highlighted in yellow in the manuscript with track changes.

**References**

1.	Kaindoa EW, Matowo NS, Ngowo HS, Mkandawile G, Mmbando A, Finda M: **Interventions that effectively target Anopheles funestus mosquitoes could significantly improve control of persistent malaria transmission in south-eastern Tanzania**. *PLoS One* 2017, **12**.

2.	Hiscox A, Otieno B, Kibet A, Mweresa CK, Omusula P, Geier M, Rose A, Mukabana WR, Takken W: **Development and optimization of the Suna trap as a tool for mosquito monitoring and control**. *Malaria journal* 2014, **13**(1):257.

3.	Mburu MM, Zembere K, Hiscox A, Banda J, Phiri KS, Van Den Berg H, Mzilahowa T, Takken W, McCann RS: **Assessment of the Suna trap for sampling mosquitoes indoors and outdoors**. *Malaria journal* 2019, **18**(1):51.

4.	Kreppel KS, Johnson P, Govella N, Pombi M, Maliti D, Ferguson H: **Comparative evaluation of the Sticky-Resting-Box-Trap, the standardised resting-bucket-trap and indoor aspiration for sampling malaria vectors**. *Parasites & vectors* 2015, **8**(1):462.
