# Supplementary material for: Fine-scale distribution of malaria mosquitoes biting or resting outside human dwellings in three low-altitude Tanzanian villages
Source: PLoS One. 2021 Jan 28;16(1):e0245750. doi: 10.1371/journal.pone.0245750 (PMC7842886; doi:10.1371/journal.pone.0245750)
Supplement: Attachment — Submitted filename: MRV_out_surveillance_PlosOne_review response.docx [file pone.0245750.s019.docx]

28^th^ December 2020

Dear Editor, PLOS ONE

**Re: Research paper re-submission (Manuscript code: PONE-D-20-16651)**

We would like to thank you and the reviewers for constructive comments and for offering us the opportunity to resubmit a revised version of our paper entitled “Fine-scale distribution of malaria mosquitoes biting or resting outside human dwellings in three low-altitude Tanzanian villages”.

Please find the detail explanation of the minor comments from Review #3 listed in point by point below. We also included further clarification with respect to the previous points, by modifying some sentence in the main manuscript, and by adding more relevant references.

Finally, we made several minor changes (and typos corrections) to further improve the reading flow. We believe our manuscript has substantially improved.

Please find our detailed point-by-point response to each reviewer’s report. We have highlighted in yellow the specific changes/additions made, in the manuscript main body.

Once again thank you very much for your support and hope to hear from you.

Yours sincerely

Arnold

[ammbando@ihi.or.tz](mailto:ammbando@ihi.or.tz)

**Response to reviewer’s comments**

**Reviewer # 3**

**Comments 1**: The authors have properly addressed the two main comments raised in the first review of the manuscript.
However, there are stlll some minor revisions that need to be done for improving the manuscript (see below).

**Response:** Thank you very much for point out this minor comments. Please find the edits listed below and highlighted in yellow in each respective page number.

**Comment 2**: Page 2, lines 49-50, write: "in the Anopheles gambiae complex and ..

**Response**: We really appreciate the additional edits. This is now added in the respective page in the main manuscript body.

**Comment 3**: Page 5, lines 162-164, write: "The Suna® traps proved to catch significantly higher number of Anopheles species in field conditions, as well as it significantly reduced entry of malaria vectors ...". Write "Anopheles" in italics (line 163).

**Response**: We really appreciate the additional edits. This is now italicized in the respective page in the main manuscript body.

**Comment 4**: Page 8, line 254, add "%" after 52.3%.

**Response**: Thank you so much reviewer for this edit. This is now added in the respective page in the main manuscript body.

**Comment 5:** Page 12, in Table 2, write "Anopheles" in italics in "Other Anopheles species" (top right side of table).

**Response**: Thank you so much reviewer for this edit. This is now italicized in the respective page in the main manuscript body.

**Comment 6**: Page 13, line 364, write "as previously described by Mala and Irungu [41]". Line 369, delete the coma between Ref No 42, 43 and (Fig. S1.11).

**Response**: Thank you so much reviewer for the edits. This is commas are now removed in the main manuscript body.

**Comment 7:** Page 14, line 386, delete "was" after "partly"

**Response**: Thank you so much reviewer for the edits. This is commas are now removed in the main manuscript body.

**Comment 8:** Page 15, lines 407-412, this paragraph is still not logical. High abundance is contradictory with the fact larval stages are washed away during the rainy season. This paragraph still needs improvement and more clarity. Line 428, delete the space after "studies".

**Response:** Thank you so much reviewer pointing out this. This is caused by heterogeneity of malaria vector densities at small scale and that there may be more factors influencing these densities than we assessed. This line is now added in the main manuscript body, in Page 15, Line 415-416.

**Comment 9**: Page 20, Ref 35, line 587, write "Anopheles" in italics. Line 597, complete Ref 40 with volume, pages, journal, etc.

**Response:** Thank you for point this out. The references are now edited.

**Comment 10:** Page 21, Ref 55, line 646, write "Anopheles" in italics.

**Response:** This is well noted. Please see it italicized in the main manuscript.

**Comment 11**: One of the 4 references mentioned in the response to reviewers is missing from the manuscript. It's the one by Mburu et al 2019.

**Response**: Thank you for this suggestion. The reference is now added in the main manuscript, Page 5, line 164
