# Supplementary material for: Fine-scale distribution of malaria mosquitoes biting or resting outside human dwellings in three low-altitude Tanzanian villages
Source: PLoS One. 2021 Jan 28;16(1):e0245750. doi: 10.1371/journal.pone.0245750 (PMC7842886; doi:10.1371/journal.pone.0245750)
Supplement: Attachment — Submitted filename: Reviwers comments_revised_060121.docx [file pone.0245750.s020.docx]

6^th^ January 2021

Dear Editor, PLOS ONE

**Re: Research paper re-submission (Manuscript code: PONE-D-20-16651R3)**

We would like to thank you and the reviewers for constructive comments and for offering us the opportunity to resubmit a revised version of our paper entitled “Fine-scale distribution of malaria mosquitoes biting or resting outside human dwellings in three low-altitude Tanzanian villages”.

Please find the detail explanation of the minor comments from Review #3 listed in point by point below. We also included further clarification with respect to the previous points, by modifying some sentence in the main manuscript, and by adding more relevant references. We have highlighted in yellow the specific changes/additions made, in the manuscript main body.

Once again thank you very much for your support and so sorry for the comments which were not well addressed during the previous review. We have now addressed all the comments which can be found in the main manuscript body.

Yours sincerely

Arnold

[ammbando@ihi.or.tz](mailto:ammbando@ihi.or.tz)

**Response to reviewer’s comments**

**Reviewer # 3**

**Comments 1**: I noticed that some comments have been taken into consideration, while others not.
The previous comments listed and sent to the authors have been made to improve the manuscript. I believe the authors forgot to integrate some of them or misunderstood some of my comments. Another possibility is that they disagreed with some of my comments, in this case an answer is expected to be provided. I’ m listing some comments again hoping this time they will all be taken into consideration.

**Response:** Thank you very much for point out additional minor comments. Please find the edits listed below and highlighted in yellow in each respective page number.

**Comment 2**: Page 2, line 49, add "the" before "Anopheles gambiae complex

**Response**: We really appreciate the additional edits. This is now added in the respective page in the main manuscript body.

**Comment 3**: Page 5, lines 162-164, improve English syntax in writing: "proved ... significantly higher ... in field conditions, ... significantly

**Response**: We really appreciate the additional edits. This is now revised in the respective page in the main manuscript body. Please see it in Page 162-164.

**Comment 4**: Page 8, line 254, delete space before %.

**Response**: Thank you so much reviewer for this edit. The space is now removed in the respective page in the main manuscript body.

**Comment 5:** Page 13, line 364, write "by Mala and Irungu". There are 2 authors only, so Mala et al is not appropriate.

**Response**: Thank you so much reviewer for this edit. The reference is now edited accordingly in the main manuscript body.

**Comment 6**: Page 15, line 418, write "Anopheline", not Anopeline".

**Response**: Thank you so much reviewer for the edits. This is now edited and can be found in the main manuscript body.

**Comment 7:** Page 20, lines 612-613, complete Ref 41 by Coosemans & Mouchet with volume, pages, journal, etc.

**Response**: Thank you so much reviewer for the edits. The reference number 41 is now edited accordingly in manuscript body.
